# Supplementary material for: Potential of Whole-Body Vibration in Parkinson’s Disease: A Systematic Review and Meta-Analysis of Human and Animal Studies
Source: Biology (Basel). 2022 Aug 19;11(8):1238. doi: 10.3390/biology11081238 (PMC9405106; doi:10.3390/biology11081238)
Supplement: Supplementary file 1 [file biology-11-01238-s001.zip › biology-1809578-supplementary.pdf]

Table S1. Assessment instruments used in the human Whole-Body Vibration studies in Parkinson's Disease populations.

Study	Outcome Measure(s)	Assessment Instrument(s)	Psychometric Properties	Population(s)[1]
1. Turbanski et al. (2005)	Postural stability	Sway reduction Narrow stance (%) Sway reduction Tandem stance (%) measured with a two-dimensional acceleration sensor	"Force plate usage provided more accurate readings when completing static balance tasks based on the visual system, whereas an inertial sensor was preferred for lower-limb tasks. The findings suggested investigation of different evaluation tool choices that can be easily adapted to suit different needs. The results for the complexity index and traditional balance indicators were comparable in their implications on different conditions" [95].	Healthy participants
2. Haas and Turbanski et al. (2006)	Motor symptoms	UPDRS-III	"The Intra-class correlation coefficients were very high: total score: 0.92; Mentation: 0.74; Activities of Daily Living: 0.85; Motor: 0.90" [96,97]<br>Adequate Face Validity [98]<br>"Studies have demonstrated that the UPDRS is sensitive to change in clinical status" [99].<br>"Based on conventional standards, the UPDRS scores were found to have excellent test-retest reliability in this sample of patients with early PD rated by academic movement disorder specialists. The ICCs for the UPDRS scores were as follows: total score, 0.92; mental, 0.74; ADL, 0.85; motor, 0.90. ICCs for derived symptom-based scales ranged from 0.69–0.88. Reliability of specific items was generally lower than for summary scales. Reliability was slightly better in patients for whom the testing interval was within 14 days" [100].	Parkinson disease
3. Haas and Buhlmann et al. (2006)	Proprioceptive performance	Minimum knee angle (°) Maximum knee angle (°) with goniometer	"Knee angles can be measured by traditional goniometers, smartphone apps are readily available and there are also purpose made digital devices. Inter-rater and intra-rater reliabilities were high for all methods (all > 0.99 and > 0.98 respectively). The digital inclinometer was the most accurate method of assessment (6° minimum significant difference). The long arm goniometer had a minimum significant different of 10°, smartphone app 12° and both visual estimation and short arm goniometry were found to be equally inaccurate (14° minimum significant difference)" [101].	Healthy young adults
4. Dincher et al. (2020)	Flexibility	Sit and Reach test (best of 3)	"Sit-and-reach tests had a moderate mean criterion-related validity for estimating hamstring extensibility (mean correlation coefficient, r= 0.46-0.67), but they had a low mean for estimating lumbar extensibility (r = 0.16-0.35)" [102].	Several populations

	Freezing	360° turn test combined (s)	Not validated as a freezing measure (is designed for dynamic balance)	
5. Dincher et al. 2021	Balance	Sway in cm on Force plate	"Primary force plate variables demonstrated excellent test-retest reliability (ICC.78 – .92). The Sensory Organization Test (SOT) and Limits of Stability test (LOS) demonstrated fair to good correlations with criterion measures, whereas the Motor Control Test (MCT) had fair correlations to balance measures only. Both SOT composite equilibrium and MCT average latency were moderately associated with disease severity" [103].	Parkinson disease
6. Kaut et al. (2011)	Motor symptoms	UPDRS-III sum score	See: 2. Haas and Turbanski et al. (2006)	Parkinson disease
7. Kaut et al. (2016)	Postural stability	Mean sway (mm) by dynamic posturography	"The Computerized Dynamic Posturography sensory organization test (CDP-SOT) is a reliable measure of balance and accurately differentiates disability status in people with MS. Collectively, the results support clinical application of the CDP-SOT as a reliable and valid measure of disease-related progression of impaired balance related to sensory integration and its utility in determining changes in balance in response to treatment. The CDP-SOT is a reliable measure of balance and accurately differentiates disability status in people with MS. Collectively, the results support clinical application of the CDP-SOT as a reliable and valid measure of disease-related progression of impaired balance related to sensory integration and its utility in determining changes in balance in response to treatment" [104].	Multiple Sclerosis
	Mobility	TUG (s)	"The Timed Up and Go (TUG) showed excellent reliability in typical adults, in individuals with cerebral palsy, in individuals with multiple sclerosis, in individuals with Huntington's disease, individuals with a stroke, and individuals with a spinal cord injury. The TUG demonstrated strong concurrent validity for individuals with stroke and spinal cord injury. Predictive validity data was limited" [105].<br><br>"The test-retest reliability of the TUG and BBS was excellent. The SEM of the TUG, and BBS were 1.16, 0.71, and 0.98, respectively. The minimal detectable change of the TUG and BBS were 3.2, 1.9, and 2.7" [106].	Cerebral palsy<br><br>Multiple sclerosis<br><br>Huntington<br><br>Stroke<br><br>Spinal Cord injury<br><br>Chronic stroke
	Fall risk	Tinetti score	"Interrater and intrarater reliability was good to excellent (intraclass correlation coefficient of .80). Tinetti Mobility Test (TMT) scores correlated with UPDRS motor scores ($r_s = 0.45$) and gait speed ($r_s = 0.53$). The sensitivity and specificity of the TMT to identify fallers were 76% and 66%, respectively. The TMT is a reliable and valid tool for assessing the	Parkinson Disease

			mobility status of and fall risk for individuals with PD" [107].	
	Gait	8MW (s)	Only valid measures of 10m walk test and 6 m walk test in PD. Not much can be found about the 8mw. But overall:<br><br>"Short-distance walking speed tests are clinically useful measures for persons with PD. The Comfortable Walking Test (CWT) and Functional Walking Test (FWT) are highly reliable and responsive to change in persons with PD. Short distance walking speed can be used to discriminate differences in gait function between persons with mild and moderate PD severity. The CWT and FWT had moderate to strong associations with other activity and participation based measures demonstrating convergent validity" [108].	Parkinson disease
	Motor symptoms	UPDRS-III score	See: 2. Haas and Turbanski et al. (2006)	Parkinson disease
8. Ebersbach et al. (2008)	Balance	Tinetti score	See: 7. Kaut et al. (2016)	Parkinson disease
	Gait	Time walk 10m (s)	See 7. Kaut et al. (2016)	Parkinson disease
	Motor symptoms	UPDRS-III score	See: 2. Haas and Turbanski et al. (2006)	Parkinson disease
	Postural stability	Mean sway (mm) by dynamic posturography	See: 7. Kaut et al. (2016)	Multiple sclerosis
9. Guadarrama-Molina et al. (2020)	Balance	Berg Balance Scale (score)	"Results support the criterion-related validity of the BBS. Its utility in other balance conditions of older adults has been established. BBS score showed significant correlations with indicators of motor functioning, stage of disease, and daily living capacity. BBS score was inversely associated with the UPDRS motor score (–.58, p = 0.005), Hoehn and Yahr Scale staging (–.45, p = 0.005), and S&E ADL Scale rating (.55, p = 0.005)" [109].<br><br>"The test-retest reliability of the TUG and BBS was excellent. The SEM of the TUG, and BBS were 1.16, 0.71, and 0.98, respectively. The minimal detectable changes of the TUG,and BBS were 3.2, 1.9, and 2.7" [106].	Parkinson disease<br><br>Chronic stroke
10. Kapur et al. (2012)	Motor symptoms	MDS-UPDRS-III score	"Concerning the validation aspects, quality of data was very satisfactory for all sections of the MDS-UPDRS, with >99 % being computable data" [110].	Parkinson disease
	Non-motor symptoms	MDS-UPDRS I score	"MDS-UPDRS Part I showed high internal consistency (Cronbach's alpha: 0.85), small floor and ceiling effects (2% floor and 0% ceiling effect), and good concurrent validity (correlation with the original UPDRS Part I: r = 0.81, p < 0.001). The standardized z-score of the	Parkinson disease

			MDS-UPDRS Part I score demonstrated high convergent validity with the composite z-score of nonmotor scales (r = 0.89, p < 0.0001)" [111].	
	Depression	BD1 score	"Confirmatory factor analysis indicated that a bifactor model with a general depression factor and three specific factors consisting of cognitive, affective and somatic showed the best fit to the data. Internal reliability was moderate to high for all subscales and for the total scale. Scores on BDI-II discriminated between clinical and general population, supporting for external validity." [112].	Several populations
	Anxiety	ISQ-anxiety	"In conclusion, the ISQ has appropriate face, content, and construct validity and is a reliable, stable and valid method to assess the past 12 month's perceived immune status" [113].	Healthy young adults
	Fatigue	FFS score	"The FFS was administered to 439 adult volunteers from the general population, 292 adults with insomnia, 132 adults with Obstructive Sleep Apnoea (OSA) and 66 adults with Chronic Fatigue Syndrome / Myalgic Encephalomyelitis (CFS/ME)<br>The FFS is a reliable and valid instrument to quantify subjective daytime fatigue. Sensitivity and specificity analyses indicate scores that best discriminate insomniacs and CFS/ME populations from a non-clinical population. However, it is proposed that the data can also be used to indicate the severity of fatigue by reference to these first two groups" [114].	General population<br><br>Insomnia<br><br>Chronic Fatigue Syndrome
11 Corbianco et al. (2018)	Recovery phase	RER calorimetry (VO2000, MedGraph, USA)	VO2000 was significantly different from the criterion Douglas Bag (DG) method for VE at 50–100 W, VO2 at rest and 100–250 W, and VCO2 at rest and 200–250 W (all, P < 0.05). The VO2000 portable metabolic system was less reliable for measuring VO2 and VCO2 and generally overestimates VO2 at most cycling work rates" [115].	Healthy adults
	Metabolic effects	Free Fatty Accids Branched-chain-AA<br><br>by venous blood and using the Free Fatty Acids, Half-micro test, Roche, Mannheim, D)	No validation studies found	-
12. Arias et al. (2009)	Gait	Gait velocity (m/s)	No validation studies found. The authors measured gait speed with no normalized test. It was calculated from the time used to cover the straight part of the task data obtained from the photocells.	-
	Stability	FRT (mm)	"Validity is regarded concurrent – good, it correlates with walking speed, tandem walk, and 1-footed stand in community dwelling elderly" [116].<br><br>"The ICC showed high reliability for the FRT	Healthy adults<br><br>Community dwelling eldery

			(GWCI, ICC = 0.83; GCI, ICC = 0.87). The absolute reliability was good: FRT, SEM = 2.96/2.29, MDC_{95} = 8.20/6.35" [117].	Older adults
	Motor symptoms	UPDRS III score	See: 2. Haas and Turbanski et al. (2006)	Parkinson disease
	Balance	Berg Balance scale (score)	See: 9. Guadarrama-Molina et al. (2020)	Parkinson disease<br><br>Chronic stroke
13. Gaβner et al. (2014)	Motor symptoms	UPDRS-III score	See: 2. Haas and Turbanski et al. (2006)	Parkinson disease
	Mobility	TUG (s)	See: 7. Kaut et al. (2016)	Stroke<br><br>Spinal Cord Injury<br><br>Chronic Stroke
	Stability	FRT (m)	See: 12. Arias et al. (2009)	Healthy adults<br><br>Community dwelling eldery<br><br>Older adults
	Gait	Step-walk turn (s)	Not validated (as far as we know). The authors developed the test. Participants must step up onto a 0.25 m high box, walk 2 m to the end of the box, turn 180 degrees, walk back, and step down. The step before stepping up and the step used to get down were placed on a force plate.	-
	Balance	One leg test (s)	"There were no significant differences between the kinematic records made by an inertial sensor during the development of the single leg stance testing between two inertial sensors placed in the lumbar and thoracic regions. In addition, inertial sensors. Have the potential to be reliable, valid and sensitive instruments for kinematic measurements during SLS testing but further research is needed" [118].	Stroke

Note: [1]Population(s) in which the psychometric properties were assessed
